# Protective effect of UDCA against IL-11- induced cardiac fibrosis is mediated by TGR5 signalling

**DOI:** 10.3389/fcvm.2024.1430772

**Published:** 2024-12-03

**Authors:** B. Reilly-O’Donnell, E. Ferraro, R. Tikhomirov, R. Nunez-Toldra, A. Shchendrygina, L. Patel, Y. Wu, A. L. Mitchell, A. Endo, L. Adorini, R. A. Chowdhury, P. K. Srivastava, F. S. Ng, C. Terracciano, C. Williamson, J. Gorelik

**Affiliations:** ^1^ National Heart and Lung Institute, Imperial College London, London, United Kingdom; ^2^ Department of Women and Children’s Health, King’s College London, London, United Kingdom; ^3^ Intercept Pharmaceuticals Inc., New York, NY, United States

**Keywords:** cardiac fibrosis, antifibrotic, TGR5, ursodeoxycholic acid, dilated cardiomyopathy, Interleukin-11

## Abstract

**Introduction:**

Cardiac fibrosis occurs in a wide range of cardiac diseases and is characterised by the transdifferentiation of cardiac fibroblasts into myofibroblasts these cells produce large quantities of extracellular matrix, resulting in myocardial scar. The profibrotic process is multi-factorial, meaning identification of effective treatments has been limited. The antifibrotic effect of the bile acid ursodeoxycholic acid (UDCA) is established in cases of liver fibrosis however its mechanism and role in cardiac fibrosis is less well understood.

**Methods:**

In this study, we used cellular models of cardiac fibrosis and living myocardial slices to characterise the macroscopic and cellular responses of the myocardium to UDCA treatment. We complemented this approach by conducting RNA-seq on cardiac fibroblasts isolated from dilated cardiomyopathy patients. This allowed us to gain insights into the mechanism of action and explore whether the IL-11 and TGFβ/WWP2 profibrotic networks are influenced by UDCA. Finally, we used fibroblasts from a TGR5 KO mouse to confirm the mechanism of action.

**Results and discussion:**

We found that UDCA reduced myofibroblast markers in rat and human fibroblasts and in living myocardial slices, indicating its antifibrotic action. Furthermore, we demonstrated that the treatment of UDCA successfully reversed the profibrotic IL-11 and TGFβ/WWP2 gene networks. We also show that TGR5 is the most highly expressed UDCA receptor in cardiac fibroblasts. Utilising cells isolated from a TGR5 knock-out mouse, we identified that the antifibrotic effect of UDCA is attenuated in the KO fibroblasts. This study combines cellular studies with RNA-seq and state-of-the-art living myocardial slices to offer new perspectives on cardiac fibrosis. Our data confirm that TGR5 agonists, such as UDCA, offer a unique pathway of action for the treatment of cardiac fibrosis. Medicines for cardiac fibrosis have been slow to clinic and have the potential to be used in the treatment of multiple cardiac diseases. UDCA is well tolerated in the treatment of other diseases, indicating it is an excellent candidate for further in-human trials.

## 1 Introduction

Cardiac fibrosis is a defining feature of maladaptive cardiovascular remodelling which occurs in a variety of cardiovascular diseases and associated conditions (1) such as dilated cardiomyopathy (DCM). The development of a fibrotic scar is detrimental to myocardial performance; reducing contractility and altering electrical conduction (2, 3)*,* leading to heart failure (HF)*.* The appearance of myofibroblasts is a crucial way-marker in the development of maladaptive myocardial fibrosis (4)*.* Myofibroblasts are a particularly active cell type, which disturb the homeostasis of cytokines, growth factors, and matrix metalloproteinases (5). This leads to excessive extracellular matrix (ECM) remodelling, facilitating formation of a fibrotic scar (6).

Evolving evidence suggests the contribution of endothelial dysfunction (7)*,* chronic low-grade systemic and myocardial inflammation to the development of interstitial cardiac fibrosis (8)*.* In the setting of chronic myocardial inflammation, activated pro-inflammatory resident macrophages (8) support the release of pro-inflammatory cytokines and chemokines including transforming growth factor-beta (TGF*β*) (9)*.* Among the multifactorial pro-inflammatory actions of TGFβ, it facilitates fibroblast activation, into myofibroblast, particularly through IL-11 signalling (10, 11). Extensive bioinformatic studies have revealed the transcriptional landscape within human fibroblasts providing previously unknown insights (10, 12, 13). These data confirm the importance of at least two gene networks which can promote fibrosis namely; TGFβ/WW Domain Containing E3 Ubiquitin Protein Ligase 2 (WWP2) stimulation of SMAD (12) and Interleukin-11 (IL-11) stimulation of extracellular signal-regulated kinase (ERK) (10). It is now clear that there are multiple fibroblast cell types and states (13), the populations of which shift depending upon the patient disease status (14). For example, it is indicated that fibroblasts isolated from the atria exhibit stronger profibrotic responses compared to ventricular fibroblasts (13). These strategies to characterise the profibrotic response offer the opportunity to better identify treatments for cardiac fibrosis, which often fail due to pleiotropic effects. Application of genomics and computational methods allow for efficient use of resources and, of particular note in this study, the ability to consider entire networks of fibrosis rather than individual pathways.

Ursodeoxycholic acid (3,7-dihydroxy-5-cholanic acid), a hydrophilic secondary bile acid, is commonly used to treat primary biliary cholangitis (15) and intrahepatic cholestasis of pregnancy (16)*.* UDCA is also beneficial in the treatment of liver fibrosis (17) and fatty liver disease in the morbidly obese (18) and those with diabetes (19). More recently UDCA has emerged as a potential treatment for Parkinson's disease (20, 21). UDCA is an agonist of multiple receptors, which is the likely reason why its mechanism of action is dependent upon the cell type, systems approaches are therefore required to investigate the mechanism. Known UDCA receptors are: farnesoid X receptor (FXR) (22), free fatty acid 4 receptor (FFAR4) (23) and TGR5 (24, 25). (25)TGR5 (gene name: *GPBAR1*) is broadly expressed in humans (and animals), including the heart (26)*.* It's expression is not limited to cardiomyocytes, but also endothelial (18) and immune cells (27), little is known about TGR5's expression in cardiac fibroblasts. Bile acid activation of TGR5 induces cyclic adenosine monophosphate (cAMP) production and also stimulates AKT (27)*,* ERK (28)*,* and NF-κB pathways (25, 29). This leads to signalling events which contribute to the regulation of basal metabolism, inflammation, and tissue regeneration*.* Current knowledge suggests that activation of the TGR5 receptor by the selective agonist INT-777 (30) reduces the expression of pro-inflammatory cytokines including TGFβ by glomerulus mesangial cells in kidney (31) decreasing renal fibrosis in diabetic mice (32) however the role of TGR5 in adult cardiac fibroblasts has not been elucidated.

The effect of bile acids upon the heart was first described by Williamson et al. in 2001, since this initial publication the mechanism of action of bile acids upon myocardial tissue has been extensively investigated (33)*.* UDCA has been identified to have cardioprotective effects against taurocholic acid-induced arrhythmia (34) and to modulate the action potential of cardiomyocytes and myofibroblasts (35, 36)*.* Several studies have proposed that UDCA and its conjugate tauroUDCA (TUDCA) may have both anti-inflammatory and antifibrotic effects in the heart (37, 38). In a mouse model of left ventricle pressure overload, induced by transverse aortic constriction, oral administration of TUDCA significantly reduced collagen deposition in the myocardium (38)*.* Interestingly, the levels of pro-inflammatory proteins (TGFβ and *p*-SMAD3) and mRNA expression of ECM proteins (Collagen 1α1 and 3α1) were decreased in the myocardial tissue of TUDCA-treated mice (38). The ability of UDCA to reduce plasma levels of pro-inflammatory cytokines was also confirmed in a small clinical study (37). In HF patients, UDCA treatment was associated with a lower concentration of soluble tumor necrosis factor α-receptor 1 (TNF*α*-1) in plasma, whereas the concentrations of TNFα-1 and interleukin-6 remained unchanged (37). In 2016, Schulz et al. reported a significant decrease of myofibroblasts in neonatal rat and human fibroblasts, cultured in hypoxic conditions after UDCA treatment (35). More recently our group has identified that UDCA, along with many other bile acids, can cause an increase in intracellular cAMP in neonatal rat ventricular myocytes (24), this effect was attributed to the activation of the receptor TGR5.

Despite promising human and cellular studies into the antifibrotic effect of UDCA, there is no evidence that UDCA is antifibrotic in adult fibroblasts and no understanding of whether it can regulate IL-11 driven fibrosis. Further to this, there is no comprehensive documentation of the mechanism of action of UDCA. In this study, we investigated the antifibrotic effect of UDCA in human and rat cell and slice culture models of cardiac fibrosis. We also performed RNA-seq upon patient fibroblasts to understand the transcriptional effect of UDCA upon two profibrotic pathways. Finally, we confirmed the antifibrotic mechanism of action of UDCA in adult cardiac fibroblasts using a TGR5 KO mouse.

## 2 Materials and methods

All reagents, unless stated, were purchased from Sigma- Aldrich (Dorset, UK), product codes are listed in their first instance. The TGR5 agonist INT-777 was provided by Intercept Pharmaceuticals, Inc. (New York, USA).

All animal experiments complied with institutional and national regulations, and approved by Imperial College London, under license by the UK Home Office, United Kingdom Animals (Scientific Procedures) Act 1986, Amendment Regulations 2012, and EU directive 2010/63/EU. Human samples were provided by the NIHR Cardiovascular Biomedical Research Unit at the Royal Brompton and Harefield NHS Foundation Trust and Imperial College London. The study was approved by a UK institutional ethics committee (NRES ethics number 09/H0504/104 + 5; Biobank approval number: NP001-06-2015 and MED_CT_17_079) and Imperial College London.

Informed consent was obtained from each patient/family involved in the study.

### 2.1 Isolation and culture of rat and mouse cardiac fibroblasts

Rat (male, Sprague Dawley, 300-350 g, Charles River) and mouse (male, C57BL/6J, WT and TGR5 KO, 20–30 g Envigo) fibroblasts were isolated by enzymatic digestion of ventricular tissue as previously described (39). The mice used in this study have been previously established as models for the study of TGR5 KO (40, 41), only male mice were used as female littermates were used in another study investigating the role of TGR5 in pregnancy. Fibroblasts were found in the supernatant after centrifugal pelleting of cardiomyocytes and were cultured for no more than 20 days (passage number <4) before cell fixation/lysis.

Rat fibroblasts were cultured in Dulbecco's modified Eagle's media (DMEM, D0819) supplemented with 10% fetal bovine serum (FBS, F9665) and 1% antibiotic-antimycotic solution (A5955), whereas mouse fibroblasts were cultured in DMEM supplemented with 20% FBS and 1% antibiotic-antimycotic, at 37°C and 5% CO_2_.

Cultures were pre-treated for 24 h with UDCA (U5127) before stimulation with 5 ng/ml IL-11 (Rat; RPA057Ra01 (Caltag Med systems), Mouse; Z03052 (Genscript)) for a further 24 h.

### 2.2 Isolation and culture of human DCM fibroblasts

Left ventricle (LV) free-wall tissue was minced into small chunks (1–2 mm^3^) and then digested in 0.05% Trypsin-EDTA (#25300054) for 2 min. Tissue chunks were then transferred into fibronectin-coated dishes (F1141) and cultured in DMEM supplemented with 20% FBS and 1% antibiotic-antimycotic at 37°C and 5% CO_2_. When confluent, fibroblasts were harvested from the dishes and used for experiments.

Cultures were pre-treated for 24 h with UDCA before stimulation with 5 ng/ml human IL-11 (PHC0115, Life Technologies) for a further 24 h.

### 2.3 Preparation and physiological monitoring of living myocardial slices

Living myocardial slices (LMS) were obtained from rat, human donor and DCM tissue, prepared and cultured in line with previous publications (42–46). Sprague-Dawley rats (300–350 g, Charles River) were anesthetised by inhalation of 4% isoflurane at 4 L/min oxygen and then sacrificed by cervical dislocation. The heart was removed from the thoracic cavity and placed in ice- cold Tyrode's slice solution (30 mM 2, 3-Butanedione Monoxime, 140 mM NaCl, 9 mM KCl, 10 mM Glucose, 10 mM HEPES, 1 mM MgCl_2_, 1 mM CaCl_2_, pH = 7.4). The left ventricle was isolated and mounted on a specimen holder coated in 4% agarose (A9539). The specimen holder was mounted in a vibratome bath (Campden Instruments, 7000 amz-2) filled with the Tyrode's slice solution. The tissue was sliced (300 µm thick) longitudinal to the fiber orientation. Slices were then attached to 3D printed T-Glase holders using surgical glue, allowing them to be mounted onto a slice stretcher which applied a fixed load upon the tissue. Slices were cultured in M-199 (with Earl's salts, M4530) supplemented with 0.1% ITS, 2% Pen-Strep, 4 nM Adrenaline, 4 nM Noradrenaline, 100 nM Dexamethasone and 2.15 nM Triiodothyronine (46) Slices were cultured at 37°C for 48 h with constant perfusion 15 ml/min and field stimulation 0.5–1 Hz (10 ms, 15 V). Co-treatments of LMS with 10 ng/ml IL-11 and UDCA were made simultaneously.

Contractility was assessed using a force transducer (Harvard Apparatus, USA). LMS were attached to the transducer using the T-Glase holders and bathed in oxygenated Tyrode's solution (37°C). The LMS was progressively stretched until a maximal isometric contraction was observed. Contractility was recorded using AxoScope software and analysed using Clampfit (both Molecular Devices, USA). Maximum contractility was normalised to control conditions of paired experiments.

### 2.4 Western blot

Cell lysates were prepared in RIPA buffer (R0278) supplemented with protease inhibitor cocktail (#11836153001) and phosphatase inhibitor (#4906845001). Tissue lysates were snap frozen and then homogenised in SB-20 (20% SDS and 0.15 mol/L Tris, pH 6.8) lysis buffer.

Samples were loaded on 8%–10% polyacrylamide gels and ran at 100 V for 1 h. Proteins were transferred to PVDF membrane (#IPVH00010) via semi-dry transfer (Trans-Blot Turbo, Bio-Rad) or wet transfer at 100 V for 1hr. Membranes were blocked with 5% skimmed milk powder (#70166) and then incubated with primary antibodies overnight (see figure legends for details). Secondary antibodies were either Alexa-fluor or HRP conjugated (see figure legend for details) and incubated with the membrane for 3 h. Blots were imaged using a Bio-Rad ChemiDoc MP.

### 2.5 Immunostaining of isolated fibroblasts

Cells were cultured on glass coverslips. After the completion of experimental protocol, cells were fixed with 4% paraformaldehyde (PFA, J19943-K2) for 15 min on ice. Cells were permeabilised with 0.05% Triton X-100 (T9284) for 15 min and then blocked for 1 h in 5% bovine serum albumin (BSA, A7906). Coverslips were incubated with primary antibody overnight, afterwards the coverslips were transferred into the appropriate secondary antibody- containing solutions for 1 h. Coverslips were then mounted onto glass slides with ProLong Gold antifade mountant with DAPI (P36941).

Images were captured using either a Zeiss LSM-780 inverted confocal laser scanning microscope or Nikon Eclipse Ti with pE-4000 light source (Cool LED) and ORCA-Flash 4 camera (Hamamatsu). Imaging was assisted by the Facility for Imaging by Light Microscopy (FILM) at Imperial College London (RE/18/4/34215). A minimum of six images were collected per experimental condition. Images were analysed with Fiji/ImageJ v1.54f. α-SMA positive cells were thresholded using two criteria: (1) mean cell fluorescence was >90% when compared to control experiments, (2) staining clearly showed the presence of filaments. Collagen I staining was determined by identifying individual cells through their DAPI and Vimentin staining. ROIs of each individual cell were then analysed for mean fluorescence values.

### 2.6 Immunostaining of LMS

LMS were washed in phosphate buffered saline (PBS) and then fixed in 4% PFA. Slices were permeabilised with 1% Triton X-100 in blocking solution (10% FBS, 5% BSA and 10% horse serum in PBS) for 3 hrs at room temperature. Primary antibodies were diluted in PBS and incubated overnight at 4°C. Slices were washed in PBS three times (30 min) and then incubated with secondary antibodies for 2 hrs at room temperature. Finally, slices were incubated with Hoechst (10 μg/ml, H3570) for 15 min and then stored in PBS at 4°C. Immunostained slices were observed using a Zeiss LSM-780 inverted confocal laser scanning microscope. Images were analysed with Fiji/ImageJ.

### 2.7 RNA-Seq

Cultured human DCM fibroblasts from left ventricle biopsies (*n* = 3), underwent three culture conditions: (no treatment, +IL-11 or +both UDCA and IL-11). Total RNA was isolated from cells with Trizol (Thermofisher Sci 15596018) and purified with Monarch RNA Clean up kit (NEB, #T2030), including treatment with DNase I to eliminate any DNA contamination. Purified RNA was given to the Imperial Genomics Facility who prepared the library and performed the sequencing experiments. Briefly, RNA quality was assessed using a Bioanalyzer (Agilent, California, USA). mRNA was selected by polyA selection and rRNA was depleted. Libraries were prepared using the TruSeq Stranded mRNACoda Sample Prep Kit (Illumina, California, USA). Sequencing depth was 20 million reads. Raw data was demultiplexed using bcl2fastq. From that point Fastqc files were generated and then assessed against standard quality metrics using FastQC, FastQ Screen and MultiQC. RNA-seq datasets were further trimmed with Fastp and aligned to the genome with STAR (47), gene counts are generated with FeatureCounts and transcripts quantified are with RSEM. Post-alignment quality metrics are collated from the post-alignment log files with MultiQC. We applied default EdgeR settings to perform differential expression analysis (48). A gene was considered to be significant if FDR < 0.05.

The Imperial BRC Genomics Facility is supported by NIHR funding to the Imperial Biomedical Research Centre.

### 2.8 Gene set enrichment analysis

Data sets were obtained from the publicly available GEO dataset: GSE97358, which was published through the research of Schafer et al. (10) and GSE133017 which was published through the research of Chen et al. (12). The IL-11 gene network was specifically constructed for this research, whereas the TGFβ/WWP2 gene network was already constructed.

We conducted gene set enrichment analysis using DAVID 6.8 (49). Ensemble gene ids were added as a gene list for Homo Sapiens with the same background. For each gene, we searched the database annotations for Gene Ontology (GO), GO_BP_Direct and in Pathways, KEGG_Pathway. We set up the database to only highlight terms with *p*-value < 0.05 (FDR and fold enrichment were also included added as additional search terms).

### 2.9 Statistical analysis of experimental data

Data is presented in text as mean ± SEM. Bar charts are presented as mean and SEM of each experimental group. All experiments were performed in biological triplicate, or greater. Datasets were tested for normal distribution using a Shapiro–Wilk test. Statistical significance of normally distributed data, unless stated, was determined by one-way ANOVA with Tukey's post-hoc test. Non-normally distributed data were assessed for statistical significance by Kruskal–Wallis (K–W) test followed by Dunn's multiple comparisons test.

## 3 Results

### 3.1 UDCA prevents the expression of cardiac fibrosis markers in wild type (WT) rat fibroblasts and rescues LMS function

**Figure 1:**
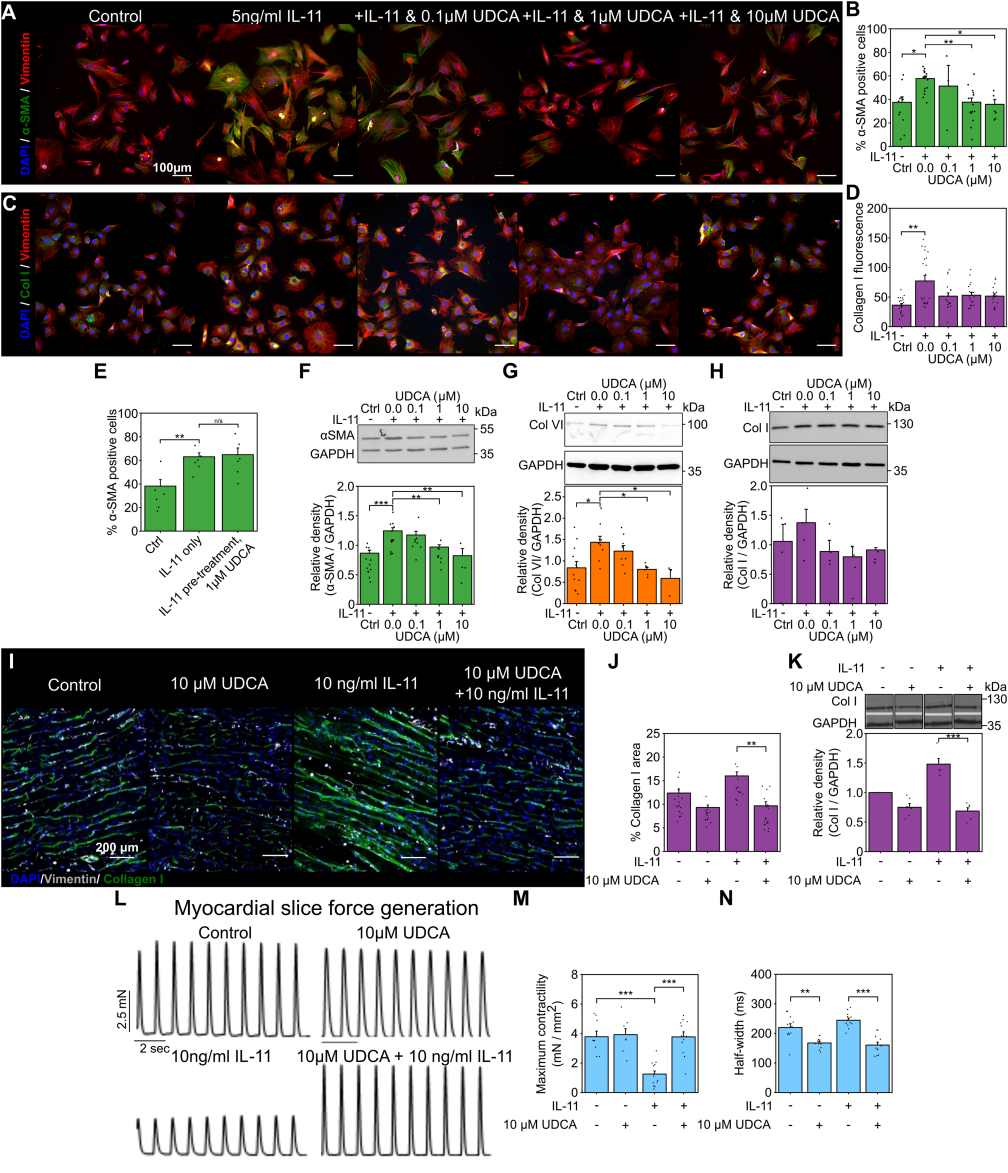
Pretreatment of cultures with UDCA is antifibrotic in WT adult rat fibroblast and LMS. **(A)** Representative images of IL-11-treated WT rat fibroblasts stained for α-SMA (1:500, M0851, Dako) green, Vimentin (1:2000, PA1-16759, Thermo) red and DAPI blue. Culture conditions are displayed above each image. **(B)** Percentage of cells positive for α-SMA staining in response to UDCA. n_experiment_ = 3–16. **(C)** Representative images of fibroblasts stained for Collagen I (1:500, ab34710, abcam) green, Vimentin (1:2000 PA1-16759, Thermo) red and DAPI blue. Culture conditions are above each image. **(D)** Mean cell fluorescence of Collagen I staining in response to UDCA. n_experiment_ = 17–22. Significance determined with K–W and Dunn's test. **(E)** Percentage of cells positive for α-SMA staining in response to IL-11 followed by 1 µM UDCA. n_experiment_ = 6. **(F)** Representative WB and quantification of WT rat cell lysate probed for α-SMA and GAPDH (1:1000, 2118, CST) in response to UDCA. n_blot_ = 4–13. **(G)** Representative WB and quantification of WT rat cell lysate probed for Collagen VI (1:500, ab6588, abcam) and GAPDH in response to UDCA. n_blot_ = 3–9. **H** Representative WB and quantification of WT rat lysate probed for Collagen I (1:500, ab34710) and GAPDH in response to UDCA. n_blot_ = 4–5. **(I)** Representative images of IL-11 treated WT rat LMS stained for Collagen I (1:500, ab34710, abcam) green, Vimentin (1:1000, PA1-16759, Thermo) grey and DAPI blue. Culture conditions are displayed above each image. **(J)** Percentage area of collagen I staining of LMS. n_experiment_ = 13–19. Significance determined with K–W and Dunn's test. **(K)** Representative WB and quantification of LMS lysate probed from Collagen I. n_blot_ = 5–6. **(L)** Representative contractile activity of WT rat LMS. Culture conditions are displayed above each trace. **(M)** Maximum contractility of LMS. n_LMS_ = 8–14. **(N)** Contractility half-width. n_LMS_ = 11–12.

We first investigated if the reported antifibrotic effect of UDCA translated to adult rat cardiac fibroblasts. Immunostaining analysis of the expression of α-smooth muscle actin (α-SMA) and collagen in fibroblasts found that pre-treatment of cultures with UDCA for 24 h, before stimulation with 5 ng/ml IL-11 reduced the percentage of α-SMA positive cells (Figures 1A,B). There was a significant reduction of α-SMA positive cells (from 57.7 ± 2.2%) when pre-treated with 1 μM (37.6 ± 3.6%) and 10 µM UDCA (35.8 ± 4.0%) (Figure 1B).

We also investigated some ECM proteins whose expression is known to be elevated in cardiac fibrosis. Collagen I staining of WT rat fibroblasts was slightly reduced by pre-treatment of cultures with UDCA, but this was not significant (Figures 1C,D). We found that when cultures were first exposed to IL-11 and then subsequently 1 µM UDCA, there was no antifibrotic effect (Figure 1E), indicating that UDCA prevents transdifferentiation of fibroblast. Western blot (WB) analysis of protein expression in WT rat fibroblasts identified significant reduction in α-SMA (Figure 1F) and Collagen VI (Figure 1G) at 1 μM and 10 µM UDCA, there was no significant change in Collagen I (Figure 1H).

We transferred our fibroblast study to a multicellular model to better understand the effect of UDCA in the myocardium. Rat LMS were cultured for 48 h [using a previously established model (46)] and then stained for collagen I (Figure 1I). Incubation of rat LMS with 10 ng/ml IL-11 significantly increased the area of Collagen I staining as compared to untreated control LMS (from 12.4 ± 0.9% to 16.0 ± 0.9%). LMS were simultaneously treated with UDCA and IL-11. We found that treatment of LMS with 10 µM UDCA and IL-11 significantly reduced the percentage area of Collagen I from 16.0 ± 0.9% to 9.7 ± 0.9% (Figure 1J). Incubation of LMS with UDCA alone had no significant effect upon collagen I staining (9.3% ± 0.5%). WB of LMS lysates showed that, co-treatment of LMS with 10 µM UDCA and IL-11 reduced the expression of collagen I (Figure 1K). The functionality of rat LMS was assessed by a force-transducer with pacing at 1 Hz (Figure 1L). Maximal contractility of LMS was reduced by IL-11 from 3.8 ± 0.4 mN/mm^2^ to 1.3 ± 0.2 mN/mm^2^. Co-incubation of the slice with 10 µM UDCA and IL-11 improved maximal contractility to 3.8 ± 0.3 mN/mm^2^ (Figure 1M). Co-treatment of LMS with 10 µM UDCA and IL-11 reduced the half-width of contractions from 244.5 ± 7.4 ms to 160.6 ± 9.9 ms (Figure 1N).

### 3.2 UDCA reduces markers of fibrosis in human DCM fibroblasts and LMS

**Figure 2:**
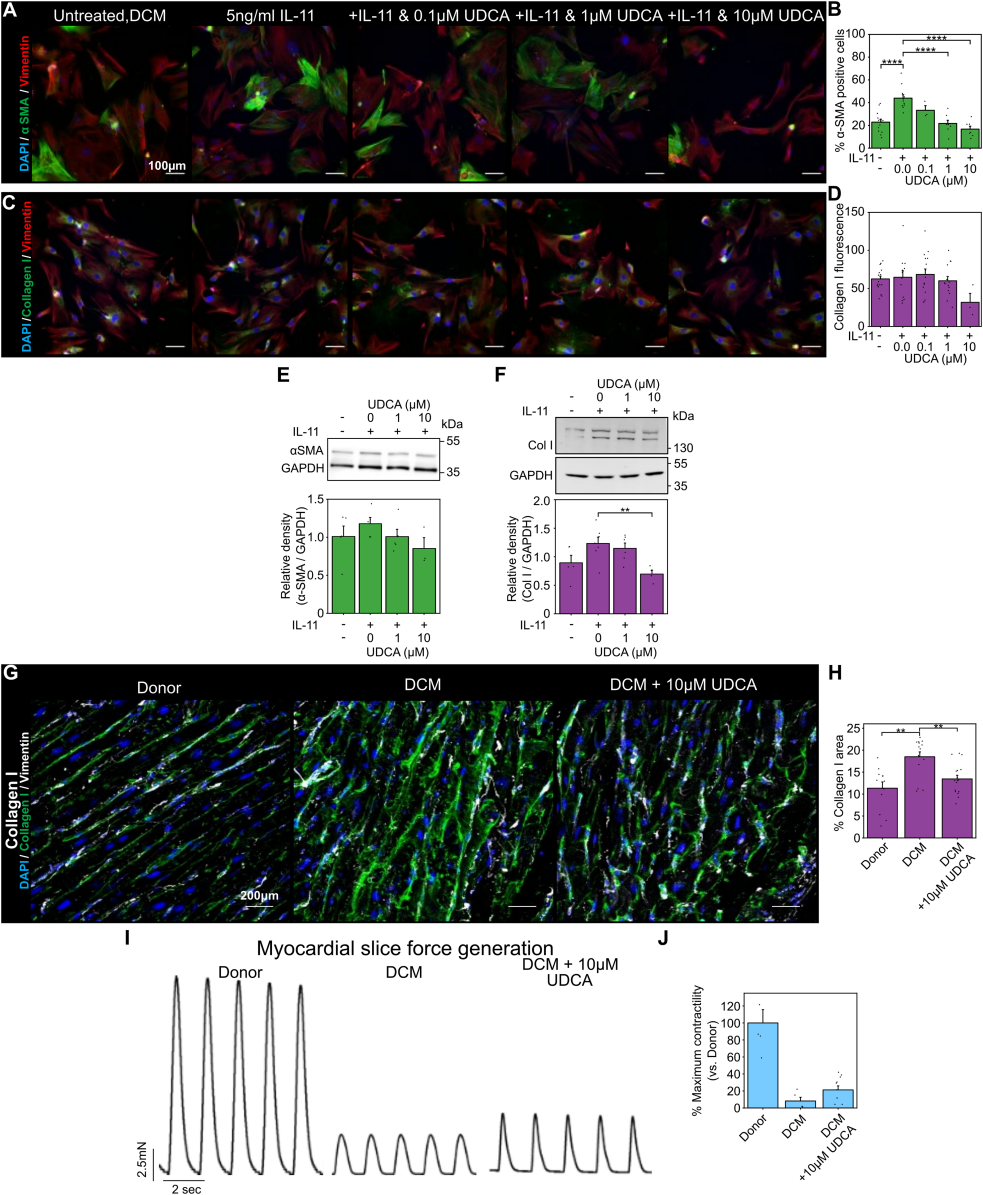
UDCA is antifibrotic in human dilated cardiomyopathy cardiac fibroblast and LMS. **(A)** Representative images of human DCM fibroblast stained for α-SMA (1:500, M0851, Dako) green, Vimentin (1:2000, PA1-16759, Thermo) red and DAPI blue. Culture conditions are displayed above each image. **(B)** Percentage of cells positive for α-SMA staining in response to UDCA. n_experiment_ = 3–13. Significance was determined by one-way ANOVA. **(C)** Representative images of fibroblasts stained for Collagen I (1:500, ab34710, abcam) green, Vimentin (1:2000, PA1-16759, Thermo) red and DAPI blue. Culture conditions are above each image. **(D)** Mean cell fluorescence of Collagen I staining in response to UDCA. n_experiment_ = 3–18. **(E)** Representative WB and quantification of human DCM cell lysate probed for α-SMA and GAPDH (1:1000, 2118, CST) in response to UDCA. n_blot_ = 3–5. **(F)** Representative WB and quantification of human DCM cell lysate probed for Collagen I (1:500, ab34710) and GAPDH in response to UDCA. n_blot_ = 4–7.**(G)** Representative images of human LMS stained for Collagen I (1:500, ab34710, abcam) green, Vimentin (1:2000, PA1-16759, Thermo) grey and DAPI blue. Culture conditions are displayed above each image. **(H)** Percentage area of collagen I staining of LMS. n_experiment_ = 12–17. Significance determined with K-W and Dunn's test. **(I)** Representative contractile activity of LMS. Culture conditions are displayed above each trace. **(J)** Maximum contractility of human LMS normalised to average human donor contractility. n_LMS_ = 8–10.

We next performed our fibroblast and LMS studies with human samples. UDCA reduced the percentage of α-SMA positive human DCM fibroblasts in a concentration- dependent manner (Figure 2A). Pre-incubation of cells with either 1 µM or 10 µM UDCA significantly reduced the number of α-SMA positive cells from 44.0 ± 3.0% to 21.7 ± 2.8% and 16.7% ± 2.6% respectively (Figure 2B). However, there was no change in collagen I staining (Figures 2C,D).

WB analysis identified a trend, but no significant reduction, in *α*-SMA expression of human DCM fibroblasts pre- treated with UDCA (Figure 2E). Counter to our fibroblast imaging studies, collagen I expression was significantly reduced following treatment with 10 µM UDCA (Figure 2F).

The area of collagen I staining in human LMS was significantly higher in human DCM slices compared to donor (18.5 ± 1.1% vs. 11.3 ± 1.4%) (Figures 2G,H). Treatment of human DCM LMS with 10 µM UDCA for 48 h significantly reduced the collagen area from 18.5 ± 1.1% to 13.5 ± 0.8% (Figures 2G,H). The contractility of LMS was also assessed (Figure 2I), LMS prepared from DCM hearts had very low contractility. The relative contractility of human DCM LMS was slightly increased when LMS were treated with 10 µM UDCA for 48 h (8.2 ± 4.4% vs. 21.3 ± 4.7% of donor LMS contractility), however this was not significant (Figure 2J).

### 3.3 RNA-Seq analysis reveals reversal of profibrotic pathways by UDCA

**Figure 3:**
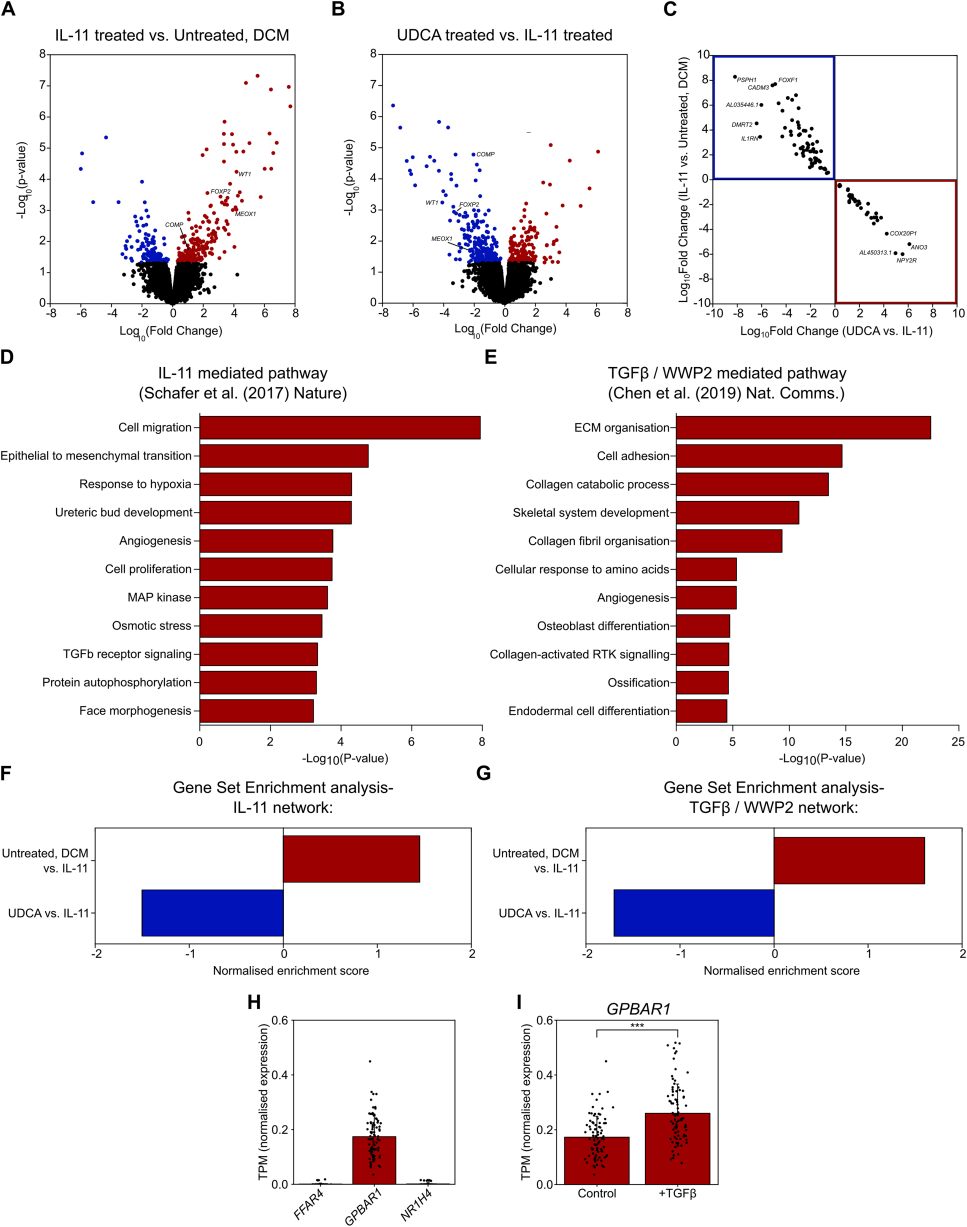
UDCA prevents profibrotic response of human DCM fibroblasts to IL-11. **(A)** Volcano plot of differentially expressed genes as determined by RNA-Seq. Conditions compared were control vs. 5 ng/ml IL-11 stimulated human DCM fibroblast. Significantly upregulated genes are marked in red, significantly down regulated genes are marked in blue. n_participant samples_ = 3. **(B)** Volcano plot of differentially expressed genes as determined by RNA-Seq. Conditions compared were 5 ng/ml IL-11 vs. 1 µM UDCA pre-treated stimulated human DCM fibroblast. Significantly upregulated genes are marked in red, significantly down regulated genes are marked in blue. n_participant samples_ = 3. **(C)** Plot of significantly regulated genes of Human DCM fibroblast. Log fold change in gene expression of IL-11 vs. Control is plotted against UDCA vs. IL-11. Genes significantly upregulated by IL-11 and down regulated by UDCA are found within the blue box (upper left quadrant). Genes significantly down regulated by IL-11 and upregulated by UDCA are found within the red box (lower right quadrant). **(D)** GO enrichment of IL-11 network in human fibroblasts (data taken from GSE97358). **(E)** GO enrichment of TGFβ/WWP2 network (data taken from GSE133017). **(F)** Gene set enrichment analysis of IL-11 network. **(G)** Gene set enrichment analysis of TGFβ/WWP2 network. **(H)** Transcripts per million (TPM) of genes encoding bile acid receptors; *FFAR4*, *GPBAR1*, *NR1H4* in human fibroblasts (data taken from GSE97358), n_participant samples_ = 84. **(I)** TPM of *GPBAR1* ± TGFβ in human fibroblasts (data taken from GSE97358), n_participant samples_ = 84, significance determined by paired Students *t*-test *p* < 0.0001.

Cardiac fibrosis is a multifactorial process, and so we performed RNA-seq to evaluate transcriptional changes associated with UDCA treatment. RNA-seq was performed upon cultured human DCM fibroblasts. Incubation of cells with 5 ng/ml IL-11 for 24 h caused a significant change [*p*-value < 0.05 or -log_10_(*p*-value)> 1.42] in the expression of 360 genes (Figure 3A). Pre-treatment of cultures with 1 µM UDCA for 24 h caused a significant change in the expression of 394 genes (Figure 3B). Profibrotic marker genes down-regulated by UDCA included; *COMP*, *FOXP2*, *MEOX1* and *WT1* [Log_10_(Fold change) = −2.04, −2.95, −2.4 and −4.08 respectively]. Figure 3C shows a plot of log_10_(fold change) of 109 genes which were significantly regulated in both IL-11-treated and UDCA-treated conditions.

We next assessed the gene ontology (GO) of two previously published datasets which investigated two pathways of cardiac fibrosis, namely IL-11 mediated (10) and TGFβ/WWP2 mediated (12). We constructed a network of the IL-11 dataset, whereas the TGF*β*/WWP2 network was already published. GO annotations of these two networks (in DAVID) found a number of pathways activated in fibroblasts during fibrosis (Figures 3D,E). Pathways activated were as expected i.e., cell migration, proliferation, ECM organisation, TGFβ receptor signaling, MAP kinase pathway and collagen fibril organisation.

Gene set enrichment (GSE) analysis of our fibroblast cultures showed that UDCA caused a significant negative regulation of both of the profibrotic pathways investigated; IL-11 (Figure 3F) and TGFβ/WWP2 (Figure 3G). Statistical significance of the network enrichment was estimated with False Discovery Rates (FDR), enrichment was considered significant where FDR < 0.05.

*In silico* analysis, revealed that *GPBAR1* (gene name of TGR5) was the most highly transcribed UDCA receptor gene in cardiac fibroblasts (Figure 3H). Expression of *FFAR4* and *NR1H4* (gene name of FXR) was negligible. We also found that the transcript per million of *GPBAR1* was significantly raised in fibroblast activated with TGFβ (Figure 3I), however in both conditions receptor expression is considered to be low.

### 3.4 Knock- out of TGR5 prevents the antifibrotic action of UDCA

**Figure 4:**
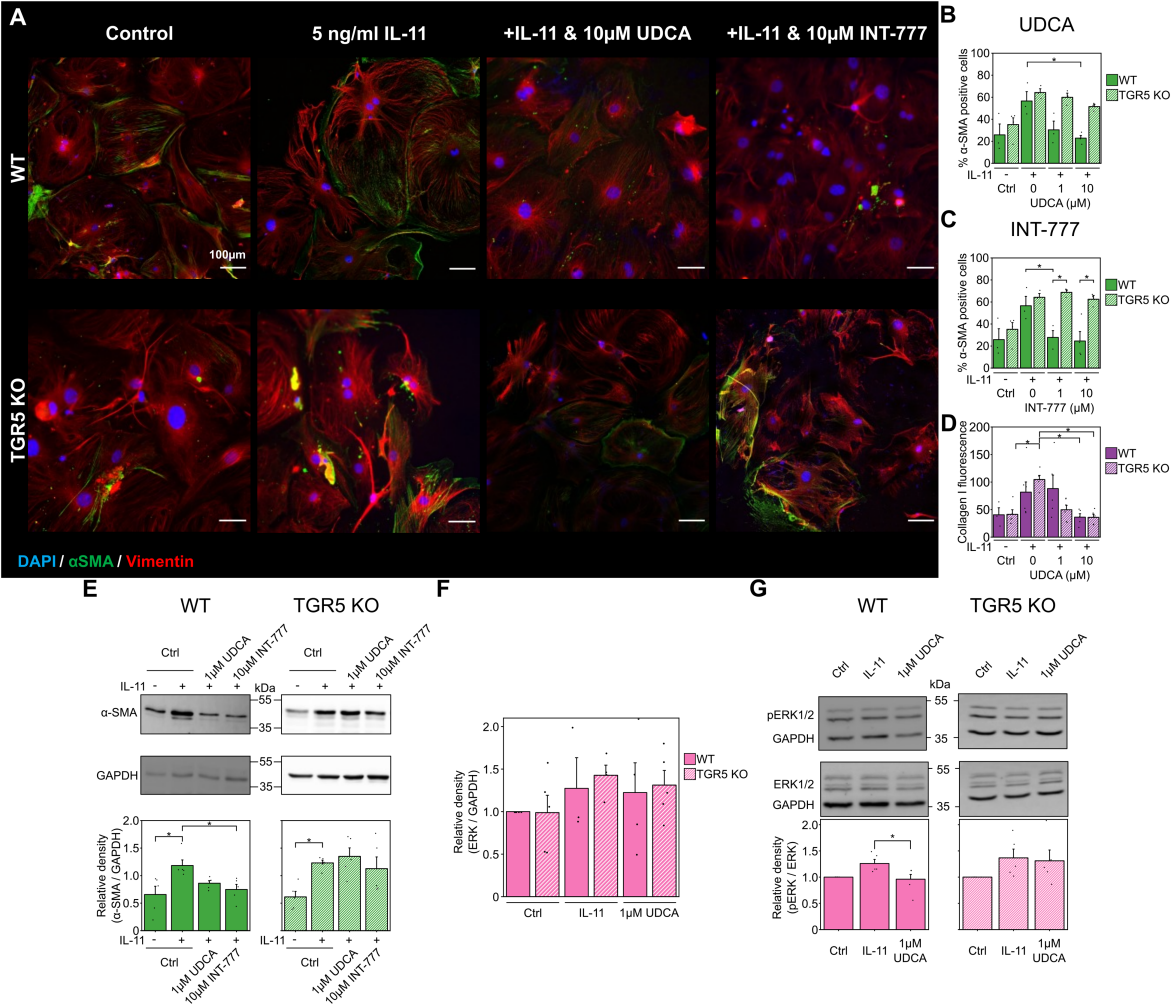
Reduction of fibrosis markers by UDCA is abolished in TGR5 KO mouse fibroblasts. **(A)** Representative images of mouse fibroblasts stained for α-SMA (1:500, M0851, Dako) green, Vimentin (1:2000, PA1-16759, Thermo) red and DAPI blue. Culture conditions are displayed above each image. **(B)** Percentage of α-SMA positive fibroblast in response to UDCA. n_experiment_ = 3–4. **(C)** Percentage of α-SMA positive fibroblast in response to INT-777. n_experiment_ = 3–4. **(D)** Mean cell fluorescence of collagen I (1:500, ab34710, abcam). n_experiment_ = 3–6. **(E)** Representative WB and quantification of mouse cell lysate probed for α-SMA and GAPDH (1:1000, 2118, CST) in response to UDCA or INT-777. n_blot_ = 4–5. **(F)** Quantification of WB for total ERK 1/2 (1:1000, 4695, CST) n_blot_ = 4–5. **(G)** Representative WB and quantification of mouse cell lysate probed for ERK 1/2 (1:1000, 4695, CST), phosphorylated ERK 1/2 (1:1000, 4370, CST) and GAPDH in response to UDCA. n_blot_ = 5–6.

As TGR5 was found to be the most highly transcribed receptor in cardiac fibroblasts (Figure 3H), we used a KO model to further probe UDCA's mechanism of action. Incubation of WT mouse fibroblasts with 10 µM UDCA significantly reduced the percentage of α-SMA positive cells from 56.6 ± 8.5% to 22.9 ± 2.3% (Figures 4A,B). There was a small reduction in the percentage of α-SMA positive cells in TGR5 KO fibroblasts pre-treated with 10 μM UDCA, likely due to off-target effects (Figure 4B). The TGR5-specific agonist, INT-777, was also found to reduce the percentage of α-SMA expressing WT fibroblasts. However, this effect of INT-777 was lost in TGR5 KO fibroblasts (Figure 4C). Mean cell fluorescence of both WT and TGR5 KO fibroblasts stained for collagen I was reduced in both cell types when incubated with 10 µM UDCA (Figure 4D). WB analysis of α-SMA reflected the findings of our imaging experiments (Figure 4E). There was no change in total ERK 1/2 expression in any conditions examined (Figure 4F). Analysis of ERK 1/2 phosphorylation identified that pre-treatment of WT fibroblasts with 1 µM UDCA significantly reduced phosphorylation (Figure 4G). These data indicate that the major receptor responsible for the antifibrotic effect of UDCA in cardiac fibroblasts is TGR5.

## 4 Discussion

This study combines RNA-seq with cellular and LMS studies, allowing for a comprehensive documentation of the effect of UDCA upon cardiac fibrosis. We have characterised this effect in rat, mouse and human fibroblasts and LMS. We show that the key receptor in mediating the antifibrotic effect of UDCA is TGR5. In this study we chose to specifically characterise the effect of UDCA upon IL-11- mediated fibrosis, which is downstream of TGFβ signalling (10) Therapies for cardiac fibrosis, which specifically target TGFβ- signalling, have had limited success (50), which is why we aimed to understand if UDCA could offer a new approach to antifibrotic therapies for cardiac fibrosis.

### 4.1 Antifibrotic effect of bile acids in adult rat fibroblasts and LMS

The antifibrotic effect of UDCA in cultured adult rat fibroblast was identified by immunostaining and WB, this is consistent with previous studies using fetal models (35). UDCA induced a significant reduction in α-SMA staining (Figures 1A,B) and expression (Figure 1F). This was not the case for Collagen I, where staining and WB analysis did not identify asignificant reduction due to UDCA treatment (Figures 1C,D,H). Interestingly, we were able to detect a reduction in expression of collagen VI, which forms part of the basement membrane, when cells were pre-treated with UDCA (Figure 1G). These data taken together indicate that pre-treatment of cultures with UDCA before stimulation with IL-11 prevents the activation of fibroblasts and therefore the emergence of myofibroblast in cultures. This correlates with similar studies investigating liver fibrosis, which attributed the reduction of fibrotic markers in hepatic stellate cells to inhibition of autophagy and the canonical TGFβ profibrotic pathway i.e., SMAD cascade (17).

Transferring these cell culture experiments to multi-cellular and hetero-cellular LMS and using a pro-fibrotic culture protocol (46), we found that collagen I staining was reduced in LMS treated with 10 µM UDCA (Figures 1I–K). We used this preparation as it offers excellent translational perspectives, linking our expression data with function. The reduction of collagen I is indicative of a reduction of ECM particularly with interstitial fibrosis, rather than focal fibrosis. Assessment of the function of the LMS was determined by force transducer, there was a clear reduction in contractility of the LMS when profibrotic pathways were activated by IL-11, co-incubation of LMS with IL-11 and 10 µM UDCA significantly increased function (Figures 1l,M). The reduction in contractile dynamics of UDCA-only treated slices (Figure 1N), compared to control, perhaps points towards some non-fibroblast mediated effects of UDCA which have been previously described. For example, it is known that UDCA can influence electrophysiological properties of the heart, and reduce ischemia-induced arrythmias (35, 36, 51, 52). In our recent publication, this was attributed to increased phosphorylation of connexin-43 proteins (52), which perhaps explains the increased contraction dynamics of slices treated with UDCA (Figure 1N). These data taken together suggest that UDCA is both anti-fibrotic and anti-arrhythmic.

### 4.2 Antifibrotic effect of bile acids in human models of cardiac fibrosis

After confirming the antifibrotic effect of UDCA in adult rat fibroblast and LMS, we turned our study focus upon human DCM fibroblast. UDCA was found to inhibit the transdifferentiation of human fibroblast into myofibroblast (Figures 2A,B). There was a significant reduction in collagen I expression, when assessed by WB. Treatment of human DCM LMS with UDCA significantly reduced the expression of collagen I (Figures 2G,H), indicating that UDCA is antifibrotic in human tissue as well as cultured fibroblast. This suggests that UDCA would limit myocardial scar proliferation *in vivo*. The reduction of collagen I in LMS did not result in a significant increase in slice contractility (Figures 2I,J). This is perhaps due to the samples available; the DCM cardiac tissue used to produce the LMS is at an end stage of heart failure and so there is a reduction in the number and function of myocytes in the slice. One may expect that reduced ECM within these LMS would result in improved diastolic function, countering the dysfunction caused by fibrosis (53). This indicates that any future treatments of heart failure involving bile acids would have a limited window if contractile function were to be maintained.

### 4.3 Role of TGR5 signaling in cardiac fibrosis

We next performed RNA-seq analysis of our cultures to better characterise the multi-factorial process of fibrosis, in these experiments performed upon human DCM fibroblast we found a number of genes to be significantly regulated when cultures were stimulated with IL-11 (Figure 3A), corroborating evidence from Schafer et al. (10). Interestingly, pre-treatment of cultures with 1 µM UDCA before stimulation with IL-11 caused an almost complete reversal of the gene expression profile (Figures 3B,C), suggesting that UDCA is antifibrotic in human DCM fibroblasts. Analysis of the previously published RNA-seq datasets using GO annotations, we found a number of pathways expected to be activated in fibroblast during proliferative fibrosis were indeed upregulated e.g., cell migration, proliferation and ECM organisation (Figures 3D,E). Gene- set enrichment analysis, against the published IL-11 and TGFβ/WWP2 datasets, showed that IL-11 enriched the profibrotic network and UDCA produced an opposing effect (Figures 3F,G). Of particular note in the network was the down- regulation of IL-11 as well as other markers of fibrosis (*POSTN* (54), *LTBP2* (55)) and inflammation (*CFH*) due to UDCA-treatment. This indicated that UDCA prevents the activation of both profibrotic pathways investigated. We assessed the abundance of UDCA receptors in cardiac fibroblasts by interrogating the IL-11 dataset (10). We found that there was a negligible level of expression for *FFAR4* and *NR1H4* whereas *GPBAR1* was positively identified (Figure 3H), indicating that TGR5 was the likely UDCA- receptor in cardiac fibroblast. The expression (TPM) of *GPBAR1* was also found to be significantly increased when human cardiac fibroblast were incubated with TGFβ, suggesting that expression of TGR5 is higher in myofibroblast when compared to fibroblast (Figure 3I).

### 4.4 TGR5 is required to prevent transdifferentiation of fibroblasts into myofibroblasts

Taking our study results together, we established that UDCA was antifibrotic in adult (human and rat) fibroblasts and LMS. We also showed that when considering the profibrotic networks at a transcriptional level, UDCA prevented the transdifferentiation of fibroblast into myofibroblast. Our analysis also suggested that TGR5 was the key UDCA receptor which could mediate this effect. We therefore isolated cardiac fibroblast from TGR5 KO mice and investigated their response to UDCA. Like our human DCM and rat fibroblast, UDCA was antifibrotic in WT mouse fibroblast (Figures 4A,B) but the effect was lost in TGR5 KO cultures. We saw a similar effect with the TGR5-specific agonist INT-777 (Figure 4C), further indicating TGR5 is the key receptor of the antifibrotic response. Our lab has previously identified that UDCA can stimulate cAMP release via TGR5 in neonatal rat myocytes (24) and that unconjugated UDCA is more potent than tauro- or glyco- conjugated UDCA. Interestingly, we found that UDCA significantly reduced the phosphorylation of ERK1/2 in WT, but not in TGR5 KO fibroblast (Figure 4G), identifying a downstream signaling pathway of TGR5 in cardiac fibroblast similar to that reported in ciliated cholangiocytes (28). These data reflect Rani et al. who, when probing whole-heart protein lysate, observed that tauro-UDCA prevented phosphorylation of ERK (38). This suggests that our proposed mechanism (based upon cellular studies) is consistent with *in vivo* work.

**Figure 5:**
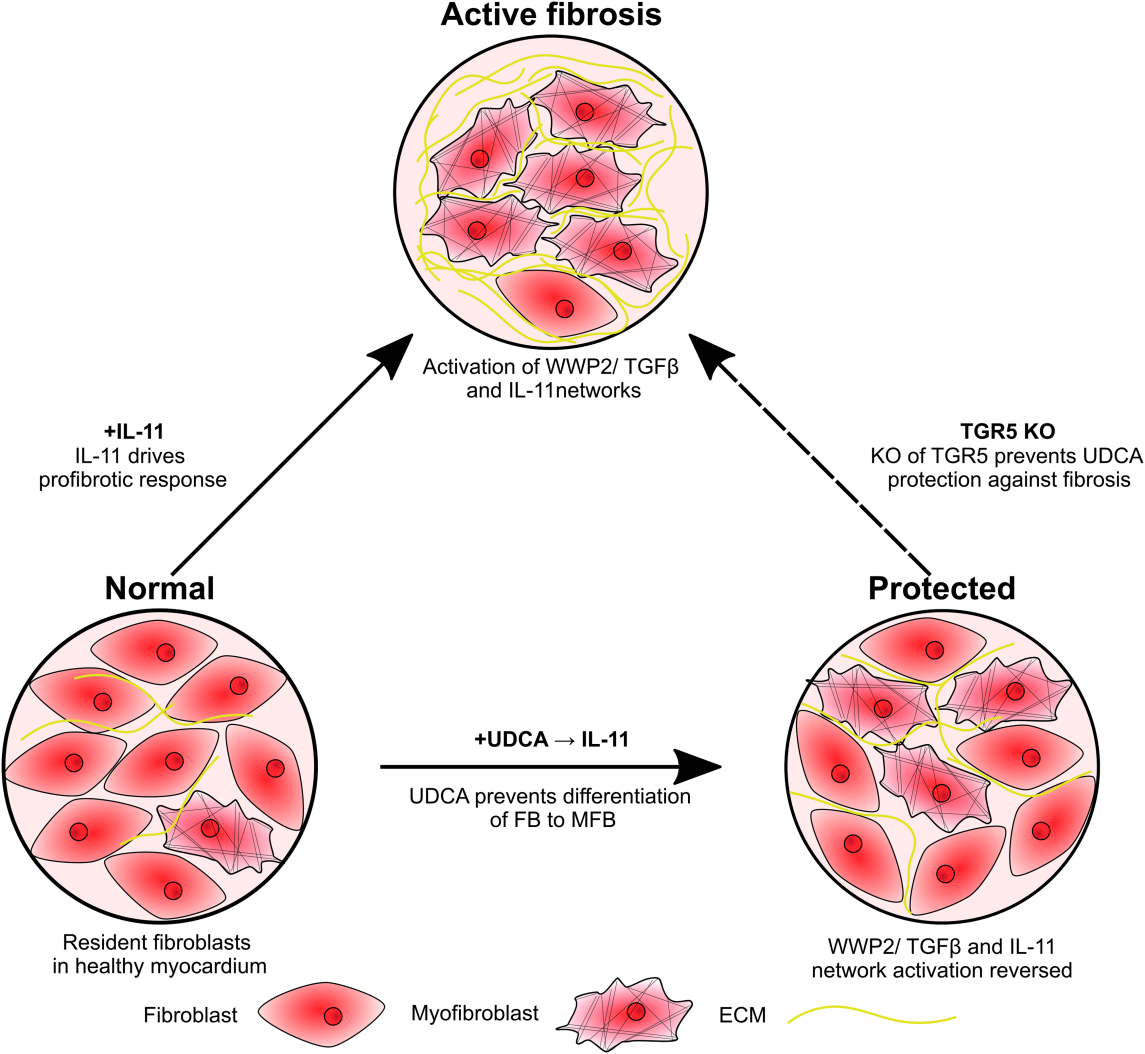
Schematic of study findings. Fibroblasts are activated by IL-11, resulting in transdifferentiation into myofibroblasts and excessive ECM production. Pre-treatment of cultures with UDCA prevents activation of the IL-11-stimulated profibrotic pathway, reducing the number of myofibroblast and ECM expression. KO of the G protein-coupled receptor, TGR5, prevents the antifibrotic effect of UDCA.

We propose that UDCA acts to prevent ERK phosphorylation, preventing the activation of the non- canonical profibrotic pathway identified by Schafer et al. (10) (Figure 5). This reveals a new pathway for the potential development of antifibrotic treatments. It may have been expected that inhibition of ERK phosphorylation would prevent inhibition of the SMAD cascade by ERK, however we did not find this to be the case, likely because our model was stimulated by IL-11 only and we pre-applied UDCA in experiments.

## 5 Conclusions

Currently there are no drugs approved primarily for the treatment of cardiac fibrosis, despite a broad range of strategies investigated (56). Although inhibitors of the renin-angiotensin-aldosterone system have been shown to have beneficial effects on interstitial fibrosis, their effects are modest (57). UDCA is safely prescribed in the treatment of primary biliary cholangitis (15) and intrahepatic cholestasis of pregnancy (58), and our data indicate that UDCA could be repurposed as a clinically valuable antifibrotic agent. There is already mounting evidence that TGR5 agonists are beneficial in patients with heart failure (37), ischemia- induced arrhythmia in rat (52) and TAC mice (38, 59). What is unclear is how the non-fibroblast-mediated effects of TGR5 signalling may affect the long-term function of the heart and other organs. Current evidence suggests that UDCA is unlikely to have an adverse effect upon the heart and indeed may contribute to better cardiac health i.e., anti- arrhythmic (52) and anti- inflammatory (37, 60). Promisingly, there were no adverse events reported during two-week administration of UDCA in adult rats (52) or to heart failure patients for 4 weeks (37). Von Haehling et al. showed that administration of UDCA can increase peripheral blood flow (37), however there was no indication if this was due to improvement of cardiac function or changes in the peripheral vasculature. Unfortunately, TGR5 is widely expressed and so direct activation of the receptor may not represent a realistic drug target specifically for cardiac fibrosis. Further investigations integrating multi-cellular methods e.g., LMS with transcriptional studies could be useful in establishing candidate genes which could be targeted in the future as cardiac- specific therapies. Another avenue would be to develop the TGR5-specific agonist INT-777 as an antifibrotic agent, removing the potential “off- target” effects of UDCA, for the treatment of cardiac fibrosis and perhaps administer this to patients with increased risk of developing fibrosis. Reducing cardiac fibrosis will likely have the benefits of improving diastolic function (61) and also reducing the incidence of ventricular arrhythmias (62), which accounts for a substantial proportion of deaths in patients with heart failure (63, 64).

## Data Availability

The original contributions presented in the study are publicly available. This data can be found here: https://www.ebi.ac.uk/biostudies/arrayexpress/studies/E-MTAB-14602.
